# Macroscopic fluorescence-lifetime imaging of NADH and protoporphyrin IX improves the detection and grading of 5-aminolevulinic acid-stained brain tumors

**DOI:** 10.1038/s41598-020-77268-8

**Published:** 2020-11-24

**Authors:** Mikael T. Erkkilä, David Reichert, Johanna Gesperger, Barbara Kiesel, Thomas Roetzer, Petra A. Mercea, Wolfgang Drexler, Angelika Unterhuber, Rainer A. Leitgeb, Adelheid Woehrer, Angelika Rueck, Marco Andreana, Georg Widhalm

**Affiliations:** 1 Center for Medical Physics and Biomedical Engineering, Medical University of Vienna, Währinger Gürtel 18-20, 1090 Vienna, Austria; 2 Christian Doppler Laboratory OPTRAMED, Medical University of Vienna, Währinger Gürtel 18-20, 1090 Vienna, Austria; 3 Division of Neuropathology and Neurochemistry, Department of Neurology, Medical University of Vienna, Währinger Gürtel 18-20, 1090 Vienna, Austria; 4 Department of Neurosurgery, Medical University of Vienna, Währinger Gürtel 18-20, 1090 Vienna, Austria; 5 Core Facility Confocal and Multiphoton Microscopy, Ulm University, N24/4105, Albert-Einstein-Allee 11, 89081 Ulm, Germany

**Keywords:** Cancer imaging, Cancer metabolism, Metastasis, Tumour biomarkers, Tumour heterogeneity, Biological fluorescence, Cancer in the nervous system, Diagnostic markers, CNS cancer, Surgical oncology, Applied physics, Atomic and molecular physics, Biological physics, Optical physics, Biomedical engineering

## Abstract

Maximal safe tumor resection remains the key prognostic factor for improved prognosis in brain tumor patients. Despite 5-aminolevulinic acid-based fluorescence guidance the neurosurgeon is, however, not able to visualize most low-grade gliomas (LGG) and infiltration zone of high-grade gliomas (HGG). To overcome the need for a more sensitive visualization, we investigated the potential of macroscopic, wide-field fluorescence lifetime imaging of nicotinamide adenine dinucleotide (NADH) and protoporphyrin IX (PPIX) in selected human brain tumors. For future intraoperative use, the imaging system offered a square field of view of 11 mm at 250 mm free working distance. We performed imaging of tumor tissue ex vivo, including LGG and HGG as well as brain metastases obtained from 21 patients undergoing fluorescence-guided surgery. Half of all samples showed visible fluorescence during surgery, which was associated with significant increase in PPIX fluorescence lifetime. While the PPIX lifetime was significantly different between specific tumor tissue types, the NADH lifetimes did not differ significantly among them. However, mainly necrotic areas exhibited significantly lower NADH lifetimes compared to compact tumor in HGG. Our pilot study indicates that combined fluorescence lifetime imaging of NADH/PPIX represents a sensitive tool to visualize brain tumor tissue not detectable with conventional 5-ALA fluorescence.

## Introduction

Although brain tumors comprise less than 2% of cancer prevalence, the affected patients suffer from severe symptoms and have a poor prognosis^1^. Despite increased efforts for improved post-operative therapy^2^, maximal safe tumor resection remains the most significant predictor of survival in the vast majority of brain tumor patients^3^. Hence, there is an urgent need for improved intraoperative tumor visualization using advanced methods such as fluorescent dyes^4^ and real-time imaging systems^5^ to achieve the surgical goal of maximal safe tumor resection.

A rather well known and tolerated fluorescent dye for neurosurgery is 5-aminolevulinic acid (5-ALA)^6^. This dye results in accumulation of fluorescing protoporphyrin IX^7^ (PPIX), an endogenous fluorophore. PPIX-based fluorescence-guidance eliminates the shortcoming of brain rearrangement in tumor resection (brain-shift) when only relying on magnetic resonance imaging (MRI)-based neuronavigation^8,9^. Originally, this 5-ALA fluorescence approach was solely used for glioblastomas^10^, but studies over the last decade have shown its additional potential for detecting anaplastic foci in suspected low-grade gliomas (LGG)^11^ as well as brain metastases (BM)^12,13^. Furthermore, visible 5-ALA fluorescence can be found in nearly every fifth low grade glioma and is there associated with a worse prognosis compared to patients where the tumor did not exhibit any fluorescence^14^. However, this fluorescence method is limited in visualizing all brain tumor tissues such as parts of the infiltration zone of high-grade gliomas (HGG), most pure LGG and a large subgroup of BM^15^.

To overcome the limitation of low PPIX visibility, spectroscopic^15^ and hyperspectral imaging^16^ for PPIX quantification have been exploited with promising results^17^. With these advanced methods it is possible to detect increased PPIX accumulations in LGG not visible to the naked eye^18^. However, these intensity-based approaches rely on the knowledge of optical tissue properties, like absorption and scattering, making the quantification more complex^19^. Additionally, the fluorescence spectra of 5-ALA labeled tumors seem to shift towards shorter wavelengths for low grade gliomas for reasons which are still under research^20^. As a potential alternative, whilst working on glioma tissue, Erkkilä et al.^21^ quantified the fluorescence lifetime of PPIX instead of its intensity, using a long working distance imaging system with similar specifications to current surgical microscopes. Although having a limited detection efficacy compared to state-of-the-art photon counting techniques^22^, this approach could show an increased sensitivity for detection of brain tumors.

Fluorescence lifetime imaging (FLIM)^22^ measures the time delay between the excitation of a fluorophore and its fluorescent response. In brief, the fluorescent molecule absorbs a photon which in return leads to an excitation from the ground state into a higher energetic state. After excitation the electrons in the molecule decay back to the ground state via multiple intermediate energy levels. While most of these transitions occur within picoseconds and only generate heat, some energy levels are more stable allowing sufficient time in the nanosecond range for fluorescence to be emitted. Due to this cascade over multiple energy levels the fluorescence is always red-shifted in relation to the excitation light which is known as the Stokes shift. Depending on surrounding molecules, different environmental conditions, or internal conversion and quenching, the electrons decay through alternative paths that alter the time delay until fluorescence is observed or even return to the ground state without fluorescence emission. Hence, the fluorescence lifetime is extremely sensitive to its molecular environment while remaining inherently independent of the fluorophore concentration^23^. This holds true for most applications where the concentration of the pure dye is relatively low compared to the solvent or environment. At higher concentrations, however, one needs to consider additional effects like self-quenching which will alter the fluorescence lifetime depending on the concentration^24^. Furthermore, the lifetime might become concentration dependent if multiple fluorophores are present or the fluorophore exists in two different aggregates with different fluorescence lifetimes. The average lifetime measured is then shifted towards the lifetime of the fluorophore or aggregate with the higher overall concentration^23^.

FLIM is extensively used to investigate endogenous fluorophores like nicotinamide adenine dinucleotides (NADH)^25^, which shows significantly elevated values in brain tumors in comparison to non-tumorous brain parenchyma^26–28^. While oxidized NAD + does not show any luminescence, free NADH emits autofluorescence with short lifetimes (400 ps). NADH is fundamental for cell metabolism and is ubiquitously involved in the glycolytic pathway as well as the citric acid (TCA) cycle and the oxidative phosphorylation in the mitochondria. Upon binding to proteins, the fluorescence lifetime of NADH increases to 1–4 ns, depending on the specific protein composition. This observation is used to monitor the reduction of NAD + during glycolysis as this increases the amount of free NADH leading to a faster fluorescence decay. Oxidative metabolism, on the other hand, is characterized by increased bound NADH prolonging the average fluorescence lifetime.

Understanding the energy metabolism in tumors^29^ is becoming increasingly important for developing therapies targeted at specific glycolytic or mitochondrial pathways^30^. Tumor cells differ from normal cells through their preferential use of anaerobic glycolysis under aerobic conditions, which is known as the Warburg effect^31^. However, several studies also observed increased oxidative phosphorylation in HGG as well as strong intratumoral heterogeneity^32–34^. Since the TCA cycle feeds heme synthesis by generating succinyl-CoA, one of the precursors that is metabolized together with glycine to 5-ALA in the mitochondria, one might hypothesize that the PPIX production might also be dependent on enhanced oxidative metabolism besides the decreased ferrochelatase^35^ found in specific brain tumors.

To address the question of PPIX fluorescence visibility and its connection to the energy metabolism of brain tumors, we performed both NADH and PPIX lifetime imaging ex-vivo on 42 human tissue samples retrieved from 21 patients with LGG, HGG and BM obtained during resection after prior 5-ALA administration. In contrast to a previous feasibility study^21^ on six patients which explored the technical feasibility of a wide-field fluorescence lifetime imaging system for intraoperative PPIX lifetime mapping, in this pilot study we extend our method to include NADH imaging and tested it on a larger study cohort (21 patients) including a broader spectrum of brain tumors. We then correlated the observed NADH and PPIX lifetimes with histology to analyze whether both fluorophores can be used together to allow improved tumor and tissue type classification only based on lifetime imaging. Finally, we used the phasor approach^36^ to study the decay dynamics of NADH and PPIX in brain tumor samples from 5-ALA guided surgery.

## Results

PPIX fluorescence visibility is highly dependent on the tumor type^15,17^. To investigate the fluorescence lifetime of NADH and PPIX for various pathologies, we analyzed a total of 42 samples obtained from 21 patients with 3 LGG (WHO II), 14 HGG (WHO III/IV) as well as 4 BM (see Table 1). Of these 42 samples, 40 specimens contained tumor tissue (n = 35) or reactive tissue (n = 5) according to histopathological analysis. In detail, in 16 samples compact tumor, in 12 samples infiltrative tumor tissue and in 7 samples tumor tissue with large necrotic areas was present. Furthermore, 5 non-tumor samples obtained during resection of BM were classified as reactive brain parenchyma due to the enhanced presence of immune cells. In contrast, in two non-fluorescing samples that were collected during the approach to deeper seated tumors no distinct tumor cells with no signs of reactive changes (no pathological brain tissue) were found.

**Table 1:** Tissue characteristics of the 42 tumor samples obtained from 21 patients.

Patient ID	Diagnosis	Samples obtained	ALA fluorescence	Tumor grade
1	Diffuse astrocytoma	TUM/TUM/TUM	− /−/ −	LGG
2	Diffuse astrocytoma	INF/INF	− / −	LGG
3	Oligodendroglioma	INF/INF	− / −	LGG
4	Anaplastic astrocytoma	TUM/TUM	− / +	HGG
**5**	Anaplastic oligodendroglioma	**NPL**	−	HGG
**6**	Glioblastoma	**INF**/TUM	− / +	HGG
**7**	Glioblastoma	INF/**NEC**	− / +	HGG
**8**	Glioblastoma	INF/**TUM**	− / +	HGG
9	Glioblastoma	INF/NEC	+ / +	HGG
10	Glioblastoma	NEC/TUM	− / +	HGG
11	Glioblastoma	NEC/TUM	− / +	HGG
12	Glioblastoma	INF/TUM	− / +	HGG
13	Glioblastoma	INF	+	HGG
14	Glioblastoma	TUM	+	HGG
15	Glioblastoma	TUM	+	HGG
16	Glioblastoma	INF/TUM	+ / +	HGG
17	Glioblastoma	NPL/TUM	− / +	HGG
**18**	Metastases (primary: lung)	**REA**/INF/TUM	+ /−/ +	MET
19	Metastases (primary: lung)	REA/NEC	+ / −	MET
20	Metastases (primary: lung)	REA/REA/NEC	− / + / −	MET
21	Metastases (primary: heart)	REA/NEC/TUM	+ /−/ +	MET
		**Total:** 42 (NPL–2; REA–5; INF–12; NEC–7; TUM–16)	**ALA positive:** 21 **ALA negative:** 21	**Partition:** LGG–3 (7) HGG–14 (24) MET–4 (11)

**Figure 1:**
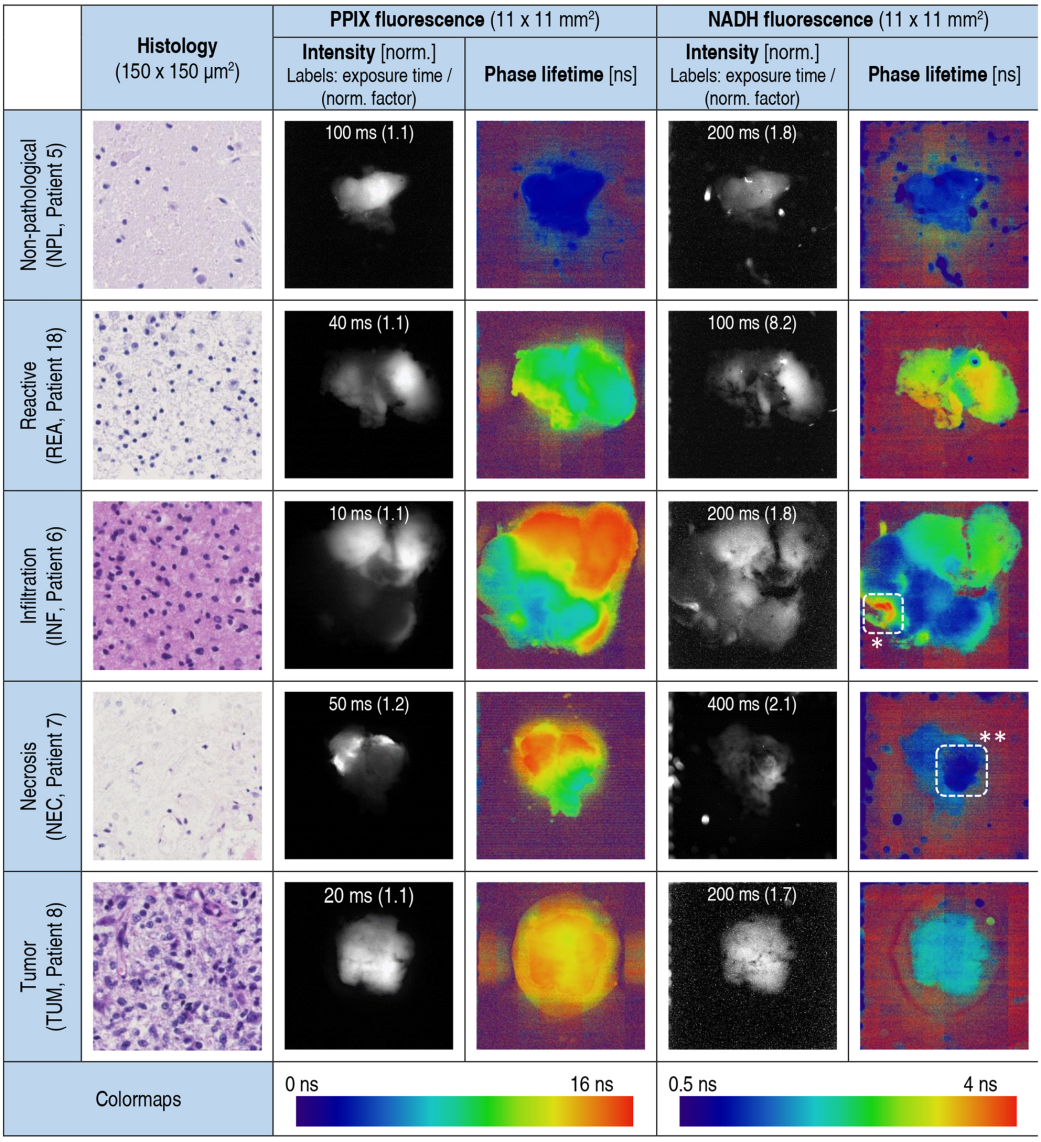
Representative NADH and PPIX fluorescence intensity and lifetime maps of tumor tissue with corresponding histology – Here we show fluorescence lifetime imaging of five representative brain tumor samples with different histopathological features. They were each obtained from a different patient as shown in Table 1. Fluorescence imaging of NADH and PPIX was performed with the pco.FLIM camera at 405 nm excitation. The fluorescence measurements (column 2–4) were performed at varying exposure times depending on the fluorescence yield (see labels in intensity images). The intensity images were then normalized for better contrast using the given normalization factor. The fluorescence lifetime maps were obtained from the phase delay relative to the modulated laser excitation. As reference we obtained histological sections for each sample investigated (see column 1). As shown malignant tissue is associated with increased NADH and PPIX fluorescence lifetime compared to non-pathological tissue. While pure fluorescence intensity imaging also shows major tumor infiltration areas, the fluorescence lifetime maps offer a more robust visualization of low fluorescing tissue. (Region of interests: *Very high NADH fluorescence lifetimes were characteristic for vessels found in histology. **NADH lifetimes below 1 ns were only found in necrotic areas).

The table shows the distribution of tissue samples obtained during fluorescence guided brain tumor resection with the patient diagnosis, the tissue histology, 5-ALA fluorescence status during surgery (visible to surgeon: +/ not visible to surgeon: −) as well as the confirmed tumor grade (LGG: low grade glioma, HGG: high grade glioma, MET: brain metastases). Bold entries specify the representative tissue samples which are shown in Fig. 1.

### Visible PPIX fluorescence is correlated to increased PPIX fluorescence lifetime

To explore the ability of FLIM overcoming the limited ability of visual PPIX fluorescence, we imaged all 42 tissue samples that were classified by the surgeon as “fluorescing” or “ALA+” in 21 cases and “non-fluorescing” or “ALA−” in the other 21 cases (see Table 1). In the following, “non-fluorescing” tissue only describes that the surgeon could not identify any fluorescence by naked eye during surgery. The samples were inspected using the fluorescence lifetime imaging system, and both fluorescence intensity as well as fluorescence lifetime maps for NADH and PPIX were acquired sequentially. Finally, the samples were sent in for histological evaluation and each sample received a unique label describing the type of tumor tissue found (see Table 1). In the following the fluorescence lifetime values were described in form of the subgroup’s median followed by the 25% and 75% quantiles in parentheses.

**Figure 2:**
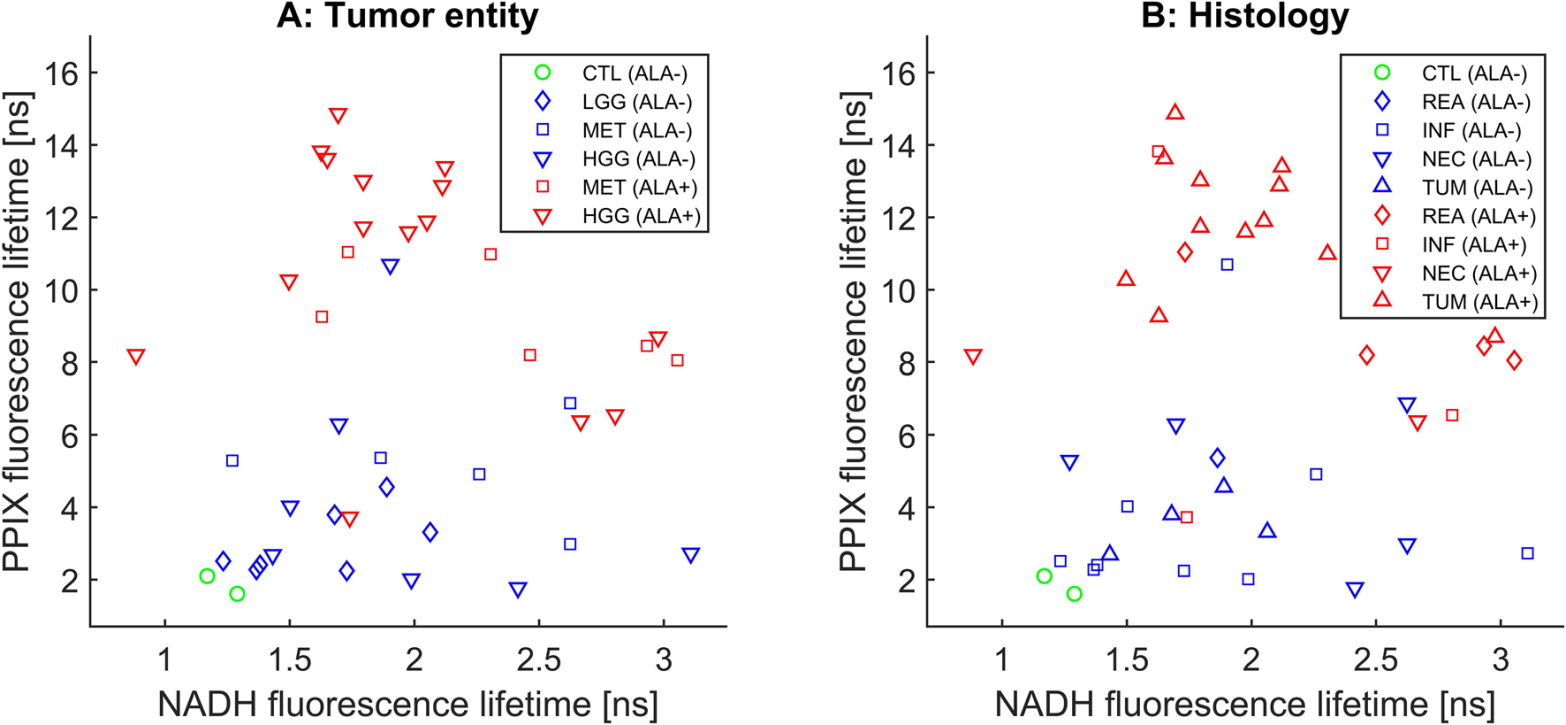
Overview of the NADH and PPIX fluorescence lifetime for each sample depending on tumor entity and tissue type—Here we show the median NADH and PPIX fluorescence lifetime for each sample. Non-pathological samples are shown in green. Samples with no visible 5-ALA fluorescence during surgery (ALA−) are shown in blue while tissue exhibiting fluorescence visible to the surgeon (ALA+) is marked in red. The symbols either define the tumor entity (**a**) or the tissue histology (**b**). Especially high grade gliomas with visible fluorescence during surgery show a rather compact cluster. Note that necrotic tissue had reduced PPIX lifetime combined with a wide range of NADH lifetimes. The sample with most necrosis showed NADH lifetimes below 1 ns suggesting glycolytic energy metabolism.

Visible PPIX fluorescence during surgery (ALA+) was correlated with an increased PPIX fluorescence lifetime of 11.0 ns (8.2; 12.9) compared to non-fluorescent tissue (ALA−) with 3.0 ns (2.3; 5.0) lifetime (p < 0.001). Although the NADH lifetime was also elevated in samples with visible PPIX fluorescence (ALA+) with 2.0 ns (1.7; 2.5), the difference to samples with 1.7 ns (1.4; 2.1), where the surgeon reported no visual PPIX fluorescence (ALA−), was not statistically significant (p = 0.14). As shown in Fig. 1, the lifetime maps offered an enhanced contrast in low intensity regions, which is most apparent for the infiltration zone with increased cell density. Very high NADH lifetime values (see Fig. 1, region of interest marked with (*) were identified as vessels in histology, while very short lifetimes below 1 ns were only observed in tissue samples with extensive necrosis (see Fig. 1, region of interest marked with **). As can be seen from Fig. 2, PPIX lifetime was also found to be reduced in necrotic areas compared to the surrounding tumor tissue. On the other hand, reactive brain parenchyma, which we only found beyond the tumor margins in brain metastases, showed long NADH lifetimes well above 2 ns. Although these samples exhibited visible PPIX fluorescence (ALA+) in over 80% of all cases, the histological evaluation did not find any tumor cell infiltration other than increased density of immune cells. The non-pathological samples yielded median fluorescence lifetimes of 1.2 ns (1.2; 1.3) and 1.9 ns (1.6; 2.1) for NADH and PPIX, respectively (Fig. 1 and Table 2). In summary, we found that fluorescence lifetimes of NADH and PPIX were highly heterogeneous between different tissue types.

**Table 2:** Descriptive statistics of the NADH and PPIX fluorescence lifetime depending on tumor entity and tissue type.

Tumor entity/grade (# samples)	NADH fluorescence lifetime (ns) median / [0.25; 0.75] quantiles	PPIX fluorescence lifetime (ns) median / [0.25; 0.75] quantiles
Low-grade glioma (n = 3)	1.7 [1.4; 1.8]	2.5 [2.3; 3.7]
High-grade glioma (n = 14)	1.8 [1.6; 2.1]	9.5 [4.0; 12.9]
Brain metastases (n = 4)	2.3 [1.8; 2.6]	8.0 [5.3; 9.1]
**Tumor tissue type**		
Non-pathological tissue (n = 2)	1.2 [1.2; 1.3]	1.9 [1.6; 2.1]
Reactive parenchyma (n = 5)	2.5 [1.8; 3.0]	8.2 [7.4; 9.1]
Infiltration zones (n = 12)	1.7 [1.4; 2.1]	3.2 [2.3; 5.7]
Necrotic areas (n = 7)	2.4 [1.4; 2.6]	6.3 [3.6; 6.8]
Compact tumor tissue (n = 16)	1.8 [1.7; 2.1]	11.3 [6.6; 12.9]

The table shows the median as well as 25 and 75% quantiles of the NADH/PPIX fluorescence lifetime values measured from the 42 samples. Note that we randomly selected 100 values in each fluorescence lifetime map to compensate for different sized samples. Pathological tissue exhibited increased fluorescence lifetimes for both NADH and PPIX compared to non-pathological tissue. Similarly, low grade gliomas showed reduced lifetimes in contrast to brain metastases and high grade gliomas.

### PPIX lifetime imaging distinguishes between tumor tissue types

**Figure 3:**
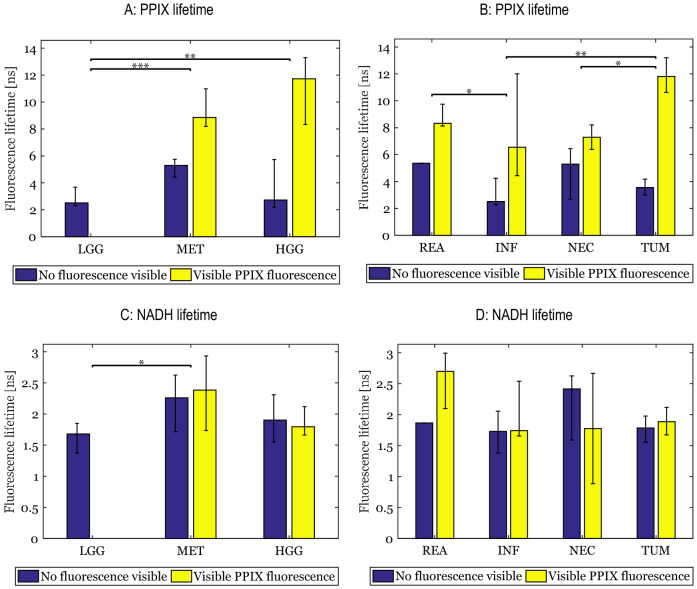
Fluorescence lifetime distribution depending on the tumor grade (PPIX: **a**; NADH: **c** and the tissue type (PPIX: **b**; NADH: **d**)—The bar height shows the median NADH/PPIX fluorescence lifetime while the error bars indicate the 25 and 75% quantiles. The PPIX fluorescence visibility was based on the surgeon’s subjective observation during resection (no visible fluorescence: ALA−; visible PPIX fluorescence: ALA+). Note that we did not include non-pathological samples due to the small sample size (n = 2) and there is no error indicated for reactive parenchyma with no visible fluorescence (ALA−) as we only found one sample with these characteristics. Significant differences between the groups are shown with an overline (*p < 0.05; **p < 0.01; ***p < 0.001) and are independent of the visual PPIX fluorescence status. Low grade gliomas showed significantly lower NADH and PPIX fluorescence lifetimes compared to brain metastases. On the other hand, metastases and high grade gliomas had fairly similar PPIX fluorescence lifetimes. Samples with visible PPIX fluorescence during surgery (ALA+) were associated with significantly elevated PPIX fluorescence lifetimes. Reactive tissue showed the highest NADH fluorescence lifetimes compared to all other tissue types.

In the next analysis, we determined whether different tissue types of brain tumors could be distinguished from each other using PPIX lifetime imaging. As can be seen in Fig. 3a and Table 2, significant differences in PPIX lifetime imaging were found between LGG and HGG (p = 0.006) as well as between LGG and BM (p < 0.001). In general, the PPIX fluorescence lifetime shown in Fig. 2 and 3b was significantly higher in compact tumor tissue compared to areas with extensive necrosis (p = 0.021). Infiltration zones were characterized by a large variance with samples ranging from values of 2.0 ns up to 13.8 ns and overall showed significantly shorter lifetimes than compact tumor tissue (p = 0.006). Similarly, reactive parenchyma was found to be significantly different to infiltration zones (p = 0.037). Note that we were also able to image non-tumorous samples with PPIX lifetimes below 2 ns which indicates that our system was sensitive enough to detect the pure autofluorescence of the tissue. Thus, PPIX fluorescence lifetime was elevated in tumor tissue and we were eager to investigate whether we could obtain similar findings in the NADH fluorescence decay.

### Altered NADH lifetimes are characteristic for reactive brain parenchyma and necrotic tumor tissue

Furthermore, we analyzed whether we could also find differences in NADH fluorescence lifetimes in our samples. According to our data (see Fig. 2, Fig. 3c and Table 2), HGG showed similar NADH fluorescence lifetimes compared to BM (p = 0.21). In contrast, LGG with 1.7 ns (1.4; 1.8) median lifetime were found to differ significantly from BM (p = 0.027). Interestingly, we could not find any statistically significant difference between the different tumor tissue types (see Fig. 3d) where all tumor grades/entities were included. However, HGG samples with necrotic areas were characterized by a wide range of NADH fluorescence lifetimes including values below 1 ns. These very short NADH fluorescence lifetimes indicate a higher ratio of free NADH relative to bound NADH which is typically associated with a glycolytic energy uptake in those areas^25^. These regions were often surrounded by compact tumor tissue with significantly elevated NADH and PPIX fluorescence lifetimes (p = 0.01) suggesting an increased oxidative metabolism. Additionally, we observed that the NADH fluorescence lifetime of reactive parenchyma with 2.5 ns (1.8; 3.0) was elevated compared to compact tumor tissue in BM with 1.8 ns (1.7; 2.1), which might be an indication for an altered, oxidative energy metabolism. However, this observation was not found to be statistically significant (p = 0.27). In general, we could demonstrate that the NADH fluorescence lifetime was significantly altered between BM and LGG as well as in necrotic areas which led us to the idea that the combined NADH/PPIX fluorescence lifetimes could be used for improved classification of the samples into the corresponding grades and tissue types.

### Combined NADH/PPIX lifetime imaging allows classification of tissue grade and types

The current practice of sending suspicious tumor tissue for intraoperative neuropathological evaluation during surgery is time-consuming and is not always available. Therefore, we used RUS boosted decision trees to classify the tumor entity/grade as well as the tissue type solely based on the NADH and PPIX fluorescence lifetime maps. To evaluate the benefit of using both NADH and PPIX fluorescence lifetime values in contrast to only relying on the pure PPIX fluorescence lifetime maps, we first performed the classification on the PPIX data alone and finally extended it to the combined NADH-PPIX dataset.

**Figure 4:**
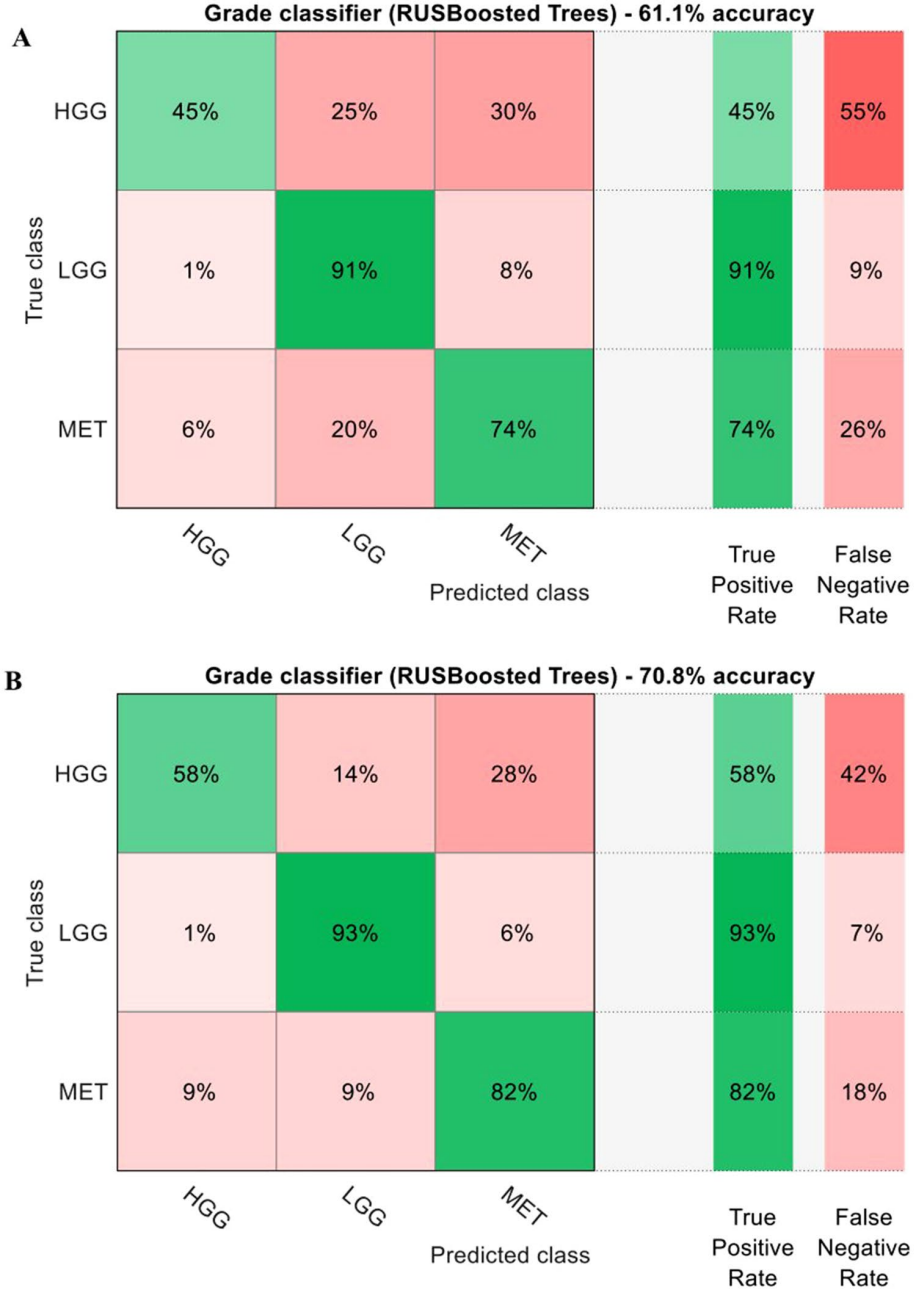
Classification of tumor entity/grade based on the fluorescence lifetime of PPIX only (**a**) and NADH/PPIX combined (**b**)—From each sample 100 fluorescence lifetime values were randomly chosen which resulted in overall 4200 datapoints. These were then classified using a RUS boosted trees classifier depending on the tumor grade. Solely relying on the PPIX fluorescence lifetime, the classifier reaches an accuracy of 61%. When adding the information of the colocalized NADH fluorescence lifetime, the accuracy reaches nearly 71%. This is mainly due to the better discrimination between brain metastases (MET) and high grade gliomas (HGG). Note that we did not include non-pathological samples due to the small sample size (n = 2).

**Figure 5:**
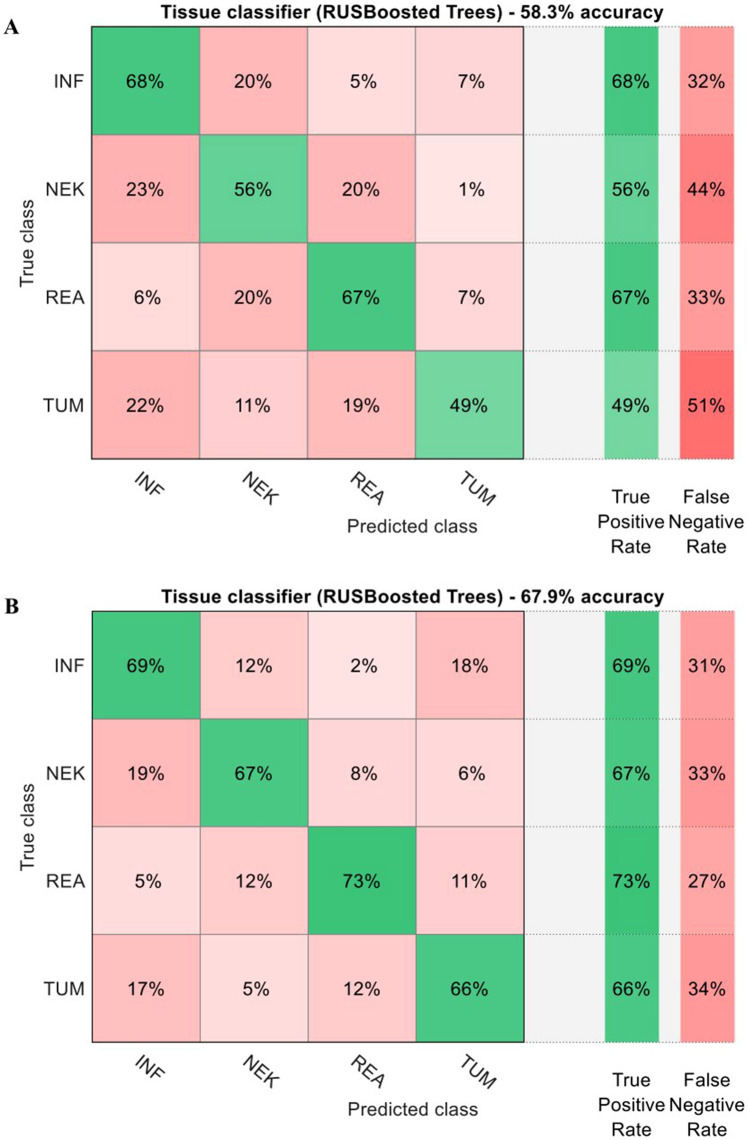
Classification of tumor tissue type based on the fluorescence lifetime of PPIX only (**a**) and NADH/PPIX combined (**b**)—Similarly to the graph shown in Fig. 4, we performed classification depending on the tumor tissue type obtained from histology. While the PPIX only classifier was already able to achieve an accuracy of 58%, the combination with NADH delivers more robust results reaching 68% correct results. Especially samples with distorted energy metabolism like reactive (REA), necrotic (NEK) or compact tumor tissue (TUM) benefit from the additional information delivered by the NADH fluorescence lifetime.

All classifiers were trained on the partitioned data (shown in Fig. 2) using a fivefold cross-validation and achieved a classification accuracy of 61.1% for the tumor entity/grade and 58.3% for the tissue types when only relying on the PPIX lifetime data (see Fig. 4a and Fig. 5a). The classification became more accurate when adding the NADH lifetime values and could improve the overall accuracy to 70.8% and 67.9% for the tumor entity and tissue type classifier, respectively (see Fig. 4b and Fig. 5b). Using this improved classifier, LGG and BM were correctly classified in 93% and 82% of all cases, respectively. HGG were mostly misclassified as brain metastases (28%), which could be expected as they share similar NADH and PPIX lifetime values. Furthermore, LGG were very rarely classified as HGG (1%). While the PPIX-only classifier didn’t perform substantially worse for detecting LGG tissue, the combined NADH/PPIX classifier mainly contributed to an improved discrimination between LGG and BM and thereby to less false negatives. This indicated that our classifier could be used for effective grading of gliomas. In the case of the tissue type classifier, necrotic areas were correctly labeled in 67% of all observations with 19% of misclassifications into infiltration zones. Although the true-positive rate for compact tumor tissue was only 66%, it had only 17% cross-talk with the infiltration zone class, which made it a sensitive method to distinguish between these different tissues. As the NADH lifetimes of necrotic and reactive tissue areas were fairly different from another, the main improvement in relation to the PPIX only classifier was found in these two classes. Hence, it would seem that combined NADH/PPIX fluorescence lifetime imagining enabled the distinction of tissue types as well as glioma grading with improved accuracy compared to relying only on the PPIX fluorescence lifetime values.

### Phasor analysis reveals bi-exponential decay of PPIX in tissue

**Figure 6:**
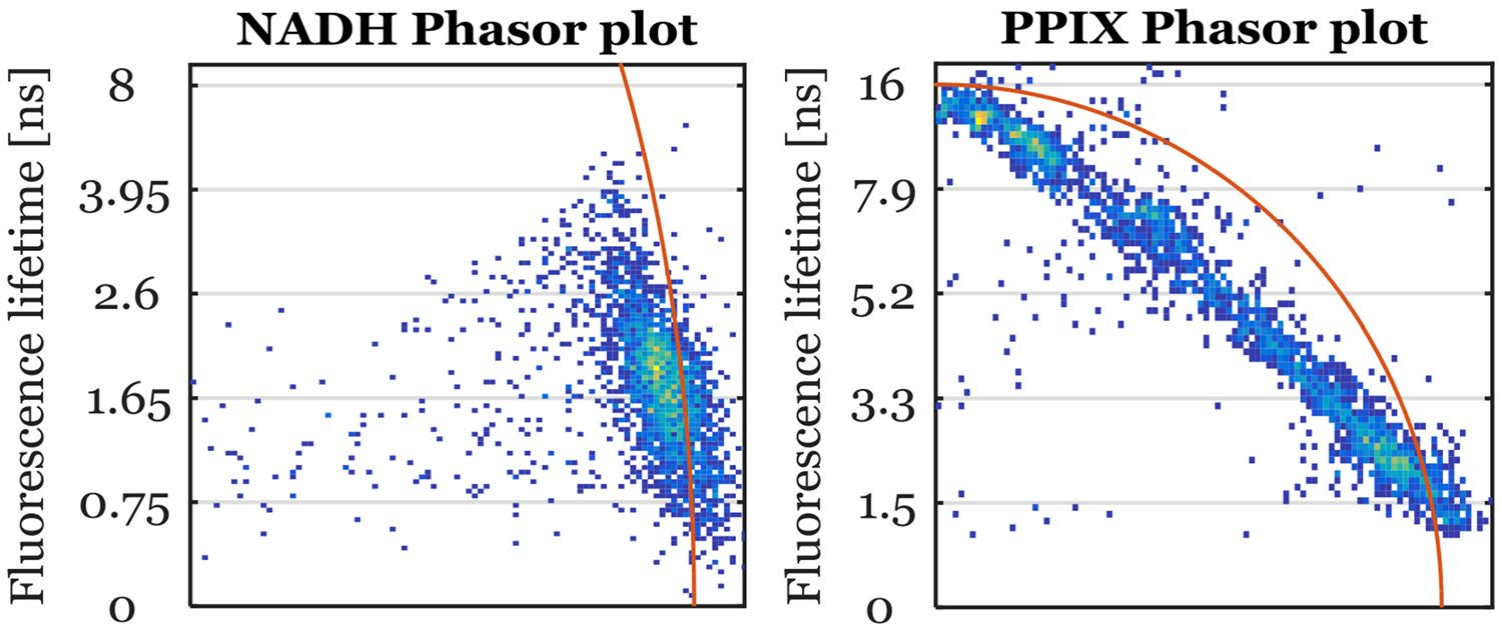
Cumulated phasor plot for NADH and PPIX fluorescence for all 42 tissue samples—The point clouds show the distribution of fluorescence lifetimes compared to the universal circle (in red). Points lying on the universal circle indicate a single-exponential fluorescence decay. However, NADH and PPIX show an elongated cloud which is indicative for a multiexponential decay. For NADH, this is due to the mixture of free and bound NADH and is well known. On the other hand, PPIX shows a clear bi-exponential decay with a long component at 16 ns and a short component below 2 ns. This indicates that the measured PPIX fluorescence lifetime of our samples is a mixture between native PPIX and tissue autofluorescence. Hence, our PPIX fluorescence lifetimes measured would be dependent on the PPIX concentration in the tissue.

While NADH fluorescence lifetime is known to be dependent on the ratio of free and bound NADH and exhibits a mostly bi-exponential decay^25^, the fluorescence lifetime of PPIX is not well understood^37^. For a more detailed investigation, we performed a qualitative phasor analysis based on the raw data of our fluorescence lifetime measurements for both NADH and PPIX. Note that the modulation frequency of 10 MHz was optimized for the long decay of PPIX. Therefore, we expected an increased variance in the NADH phasor plots (see Fig. 6).

PPIX fluorescence showed a linear relationship between a fast lifetime component with similar values to the non-pathological samples with 1.9 ns and a longer decaying component with a lifetime of 16 ns which is usually found for pure PPIX in solvent^37,38^. This observation revealed that the measured fluorescence lifetime of PPIX in tissue is, in fact, a bi-exponential decay and would, thus, be dependent on the concentration ratio of the longer and faster decaying component. Taking into account the exposure times shown in the fluorescence intensity maps in Fig. 1, there is also evidence that an increased PPIX lifetime is associated with increased PPIX fluorescence intensity. On the other hand, the phasor plot of NADH resembled an elongated ellipse with one of the focal points lying on the universal circle. This point indicated the presence of short lifetime component below 1 ns which would likely be free NADH (400 ps^25^). The other focal point lied within the circle and had a larger variance as expected. These longer fluorescence lifetime components are most likely due to various proteins in the brain tissue binding to NADH and altering its fluorescence lifetime.

Hence, our analysis revealed that both NADH and PPIX fluorescence decayed with short and long lifetime components. While the bi-exponential decay of PPIX was fairly evident, the decay of NADH we measured might also include more than two contributing components.

## Discussion

Although PPIX fluorescence-guided neurosurgery has been widely adopted within the last two decades^9^, the method of evaluating the fluorescence by the naked eye has remained unchanged. This approach, however, limits the detection of small PPIX accumulations in LGG and infiltration zones of HGG. Here we show for the first time using a novel time-of-flight camera based time-resolved fluorescence imaging system that specific brain tumors, including LGG, with no visible 5-ALA fluorescence during resection (ALA−) exhibited elevated PPIX fluorescence lifetimes compared to values of physiological brain parenchyma measured and found in literature^39^. Furthermore, NADH fluorescence lifetime was found to be significantly altered in necrotic tumor tissue compared to compact tumor tissue. By combining both NADH and PPIX fluorescence lifetime information, we could show that different tumor entities as well as tumor tissue types have distinct NADH/PPIX fluorescence lifetime features enabling an improved classification. Finally, we could demonstrate that the observed PPIX fluorescence lifetime is, in fact, a weighted sum of long lifetime component at around 16 ns, most likely due to pure PPIX, and a shorter lifetime component below 2 ns which was not known so far. To our knowledge, we are the first to obtain parallel NADH and PPIX fluorescence lifetime measurements using an imaging system offering a macroscopic square field of view (11 mm) combined with a suitable working distance (250 mm) and a real-time preview within seconds for future simultaneous resection. Therefore, our results strongly indicate that FLIM of NADH and PPIX is technically feasible and could be used for intraoperative guidance as well as tumor grading.

Our observations on NADH fluorescence lifetimes in brain tumors were in good agreement with the measurements of Sun et al. on glioblastomas (lifetimes ranging from 1.2 in cortex up to 2.6 ns in tumor)^26^. Similarly, our PPIX fluorescence lifetimes were in the range between 1.9 ns for non-pathological tissue up to almost 16 ns in high grade tumors which reflects the large variability of PPIX fluorescence lifetimes reported in literature^21,38^. As both BM and HGG are rapidly proliferating tumors, it is likely that the increased NADH lifetimes compared to LGG are indicative for their mutated and upregulated mitochondrial metabolism. We also observed NADH lifetime values in HGG and BM below 1 ns representative for glycolytic energy uptake. It is, however, known that glioblastomas switch between glycolytic and oxidative metabolism depending on the availability of oxygen^33,40^. As we did not observe any NADH lifetimes below 1 ns in LGG, these less aggressive tumors do not seem to use anaerobic/glycolytic pathways. Therefore, we hypothesize that as LGG form, the oxidative metabolism increases first which subsequently generates more succinyl-CoA followed by 5-ALA in the mitochondria. This leads to an upregulation of the heme synthesis generating more PPIX. Our hypothesis would imply that the PPIX in LGG is mainly produced by endogenous ALA in contrast to high-grade tumors where the administered ALA can penetrate through the ruptured brain-blood barrier and short circuit the TCA cycle. This would confirm the findings of Yang et al.^35^ that isocitrate dehydrogenase (IDH) mutation, which is mainly found in LGG, leads to an increased accumulation of TCA cycle metabolites and enhanced production of mitochondrial NADH. In contrast to the mutated ferrrochelatase in HGG, as LGG are still able to convert PPIX into heme, the elevated endogenous ALA would be quickly metabolized and would therefore only generate a minor elevation of PPIX levels in the tumor cells. We suspect this could be a reason for why LGG do not exhibit visible PPIX fluorescence (ALA+) and have much lower PPIX fluorescence lifetimes compared to HGG. In the future, we thus want to correlate the NADH and PPIX fluorescence lifetime with the IDH mutation status to further understand this ambiguity and investigate whether this genetic factor can be predicted.

We also found high NADH and PPIX lifetime values in reactive brain parenchyma of BM which did not show tumor cell infiltrations. The increase in PPIX lifetime confirms the reports of Kamp et al.^12^ and Utsuki et al.^13^ on visible PPIX fluorescence (ALA+) beyond the tumor margins in these secondary tumors. Furthermore, the high NADH lifetime indicates an enhanced oxidative energy uptake in these areas. Although the NADH lifetimes in compact tumor tissue of BM were reduced compared to reactive parenchyma, they also exhibited rather long NADH lifetimes which might be linked to the highly proliferating nature of these tumors. As we observed that the PPIX fluorescence visibility during surgery was linked to enhanced PPIX fluorescence lifetime combined with the fact that the lifetime showed a bi-exponential decay, it is likely that our measured lifetimes are dependent on the concentration of PPIX in the tissue. While we hypothesized in our previous publication^21^ that quenching would be the main driver for the reduction of PPIX fluorescence lifetime in tissue, our observations suggest that the ratio between the native tissue autofluorescence and the PPIX fluorescence and thus the PPIX concentration in tissue itself is the key to explain the high variability in the measured fluorescence lifetimes. However, as discussed by Rueck et al.^41^, depending on different enzymes which control 5-ALA metabolism, shorter fluorescence lifetimes of other porphyrins as Uroporphyrin and Coproporphyrin could also explain this observation. This also includes photoproducts of PPIX which might be produced during longer illumination in surgery and have fluorescence lifetimes in the range of 2–4 ns^37^. Furthermore, the detection bandwidth from 580–730 nm is rather large which could also include additional endogenous fluorophores besides porphyrins like, for example, lipofuscin. The fluorescence lifetime of lipofuscin has been shown to consist of a short component at 390 ps and a longer component at 2.2 ns^42^ and would thus be in the same range of values that we observed for non-pathological tissue. Spectroscopic time-resolved measurements would be the future tool of choice to analyze the exact underlying mechanism of our measured PPIX fluorescence lifetimes.

The findings of this pilot study have to be seen in light of four main limitations. (1) First, our study cohort is highly heterogeneous with a high number of HGG samples compared to LGG and BM. This was expected as the primary indication of 5-ALA fluorescence guided surgery are HGG and therefore resection of LGG and BM under 5-ALA fluorescence guidance are performed less often. While our study population reflects this behavior, it overall leads to highly imbalanced classes of data which is the main reason for the poor accuracy of our classifiers with regard to the determination of the tumor type and grade. We already partly tackled this problem by employing specialized classification algorithms which handle class imbalance. Otherwise, most classifiers would completely misclassify the smallest group, as the largest group would always be correctly detected, and therefore the accuracy would be maximized. The lifetime heterogeneity can also be seen within individual samples, especially for tumor tissue that was later found to be of infiltrative nature. Although these specimens showed also signs of non-pathological and compact tumor tissue, we labeled these samples according to the main contributing tissue type. Therefore, the median fluorescence lifetime values might deviate from more homogeneous samples and thus lead to a less significant difference between the tissue type classes. Future studies will need to address these limitations by only relying on fairly homogeneous samples or creating co-registered histopathological slices for each individual sample. However, this will not resolve the fact that the primary indication for 5-ALA fluorescence guided surgery currently remains HGG. For this point, larger studies are needed to collect sufficient number of samples from other tumor types which might take several years. In contrast, this pilot study was intended to evaluate the feasibility of this method rather than providing clinical evidence for the efficacy of NADH/PPIX fluorescence lifetime imaging. (2) In addition, we only included two samples with non-pathological brain parenchyma and thus this study lacks a reliable control group. Although the measured lifetime values of these two non-tumorous samples were in agreement with literature stating an average NADH fluorescence lifetime of 1.3 ns^26,43^ as well as 1.8 ns for PPIX in physiological brain tissue^39^, we did not include these specimens in our statistical analysis due to the small sample size. Therefore, future series are needed with a larger number of tissue samples in order to generate a statistically valid control group. Note that this step is non-trivial as non-pathological tissue is mostly obtained from the access route to deeper seated tumors and removal of tissue for research purpose is minimized due to ethical reasons. Therefore, collecting a sufficiently large control group might take several years, which was not the intent of this first pilot study. Nonetheless, we are targeting these issues in an extended, on-going clinical study. (3) Third, our fluorescence lifetime imaging system was primarily designed to optimally resolve the long fluorescence lifetime of PPIX and the use of our 405 nm laser is less optimal for NADH imaging. Conventionally NADH is excited at 375 nm^32,44^ which maximizes the fluorescence yield and prevents the excitation of other endogenous fluorophores like flavin adenine dinucleotide (FAD). However, several studies have shown that NADH remains the main fluorescence contributor in tissue when using one-photon excitation at 405 nm^45,46^ or two-photon excitation at 800 nm^47,48^ despite additional excitation of FAD. Although our results revealed reasonable lifetime values for NADH and, thus, confirmed that we mainly excited NADH, switching to a higher modulation frequency (> 40 MHz) as well as to shorter excitation wavelengths (< 400 nm) would have significantly enhanced the lifetime precision for NADH. However, our study specifically employed a blue-violet laser as this wavelength range is equivalent to the blue-light illumination found in commercial neurosurgical microscopes with 5-ALA fluorescence option, thus, simplifying the regulatory constraints towards an investigational device for future in vivo use. In addition, the time-of-flight camera in our system requires an additional calibration step using a reference target when changing the modulation frequency. Therefore, we would have needed to reposition the samples between the PPIX and NADH fluorescence lifetime acquisition which would have hindered the co-registration between both acquisitions. (4) Finally, our field of view is certainly macroscopic as found in similar imaging systems^44^, however, it is still rather small considering its potential application to image the whole surgical cavity. To increase the field of view even further one could either develop a camera with a larger sensor or reduce the focal length of the camera lens. In the latter case, the light throughput should optimally be identical to the current setup. For example, a 50 mm f/1.0 would be equivalent in throughput to the current 100 mm f/2 while doubling the field of view at the same working distance.

To conclude, we presented a first pilot study on combined macroscopic fluorescence lifetime imaging of PPIX and NADH enabling enhanced brain tumor visualization as well as reliable assessment of tumor type and grade. PPIX fluorescence lifetime imaging enabled a more robust visualization of infiltration zones and low grade gliomas in general, whilst the time-resolved imaging of NADH offered additional insight to distinguish necrotic tissue areas. In comparison to pure intensity imaging, the fluorescence lifetime offered better contrast in highly heterogeneous tissue areas like infiltration zones and could thus become a valuable addition to conventional 5-ALA fluorescence guided surgery. While further in vivo studies are needed to confirm the clinical benefit of FLIM to delineate tumor borders, our results pave the way towards future intraoperative fluorescence lifetime imaging of PPIX and NADH for improved resection of brain tumors.

## Materials and methods

### Study design

The investigation of the fluorescence lifetime of NADH and PPIX was performed on freshly resected tumor samples ex vivo according to national regulations as approved by the ethics committee of the Medical University of Vienna under the approval number EK419/2008—Amendment 04/2018. Adult patients with either diffuse gliomas (WHO grades II–IV) or BM undergoing tumor resection after preoperative 5-ALA administration (20 mg/kg bodyweight; approximately 3 h before anesthesia) were included in the study after obtaining informed consent. During surgery, a state-of-the-art neurosurgical microscope with violet blue illumination was used for excitation of PPIX fluorescence and tissue samples in the suspected tumor region as well as during approach to deeper located tumors were safely collected. The fluorescence status of each collected tissue sample was subjectively classified by the neurosurgeon as visible (ALA+) or no visible fluorescence (ALA−) based on his observation by naked eye. The collected tissue samples were stored in artificial cerebrospinal fluid to maintain cell viability^49^. Although NADH fluorescence lifetime has been shown to be fairly constant within the first 8 h after resection when stored in nutrient solution^50^, we immediately transferred the samples to the microscopy lab for imaging within an hour after resection to avoid any degradation of the cell metabolism. After imaging, the samples were directly transferred to the neuropathology department for histological evaluation. The pathologists established the tumor diagnosis for each patient according to the current World Health Organization (WHO) criteria^51^. The samples were further classified into (i) compact tumor tissue, (ii) necrosis, (iii) infiltration zones, i.e., brain parenchyma with diffusely infiltrating tumor cells, (iv) reactive parenchyma, i.e., brain parenchyma with reactive changes such as astrogliosis or significant macrophage infiltration but without clear-cut tumor cell infiltration and (v) non-pathological parenchyma, i.e., brain parenchyma without significant reactive changes (other than mild edema) and without tumor cell infiltration.

### Fluorescence lifetime imaging

Imaging was performed on a custom built long working distance (250 mm) fluorescence lifetime microscope using a modulated 405 nm laser and a dedicated time-of-flight camera (pco.FLIM, pco AG, Germany) as previously reported by Erkkilä et al^21^. The camera was controlled by a proprietary microscopy software (NIS Elements, Nikon Instruments Europe BV, Netherlands) with an integrated pco.FLIM plugin offered by the camera manufacturer. The large field of view of 11 × 11 mm^2^ as well as the extended working distance were specifically chosen to meet the specifications of commercial neurosurgical microscopes. In brief, the modulated continuous wave laser excites the fluorophores like NADH and PPIX which respond by emitting fluorescence at the same frequency but with a lifetime dependent phase delay. The camera detects this phase delay by storing the generated photo-electrons in two separate charge bins depending on whether the laser is on or off over the full exposure time. The ratio between both bins can then be used to reconstruct the phase delay as well as the amplitude ratio (known as modulation depth) between excitation laser and the fluorescence signal. The camera acquires a total of 16 frames at different time delays of the excitation laser to reconstruct the fluorescence phase delay. A reference target with a known lifetime is then used to reference the system and convert the phase into lifetime maps. Note that we only computed the fluorescence lifetime from the phase delay and did not include the modulation depth. The fluorescence was filtered using a (466 ± 20) nm and (665 ± 75) nm bandpass filter for NADH and PPIX, respectively. Both PPIX and NADH lifetime maps were acquired sequentially at the same position by switching the emission filter in front of the camera. The modulation frequency of the laser was fixed to f = 10 MHz which is optimized for ${\tau }_{opt}=1/(2\pi f)=15.9$ ns^52^ as PPIX exhibits a native lifetime of around 16 ns^37,38^. An acquisition including processing took maximum 7 s for the longest exposure time setting of 400 ms at an incident laser power of 50 mW/cm^2^. The exposure time was set individually for each sample and fluorescence channel (NADH/PPIX) and was optimized in the way to integrate as long as possible while avoiding saturation of single pixels. To validate the system a cuvette with PPIX dissolved in DMSO (1 µg/ml) was measured and a fluorescence lifetime of (16.4 + /−1.0) ns was obtained, which was in good agreement with literature^21,38^.

### Post processing for statistical analysis

As most samples were smaller than the full field of view, we manually segmented the areas containing tissue in both NADH and PPIX lifetime maps with an image processing program (ImageJ). All values outside the segmented area did not contribute to the further analysis. These segmented maps were then imported into MATLAB where a mask was applied to remove outliers. This consisted of limiting the lifetime to meaningful values (NADH < 5 ns; PPIX < 17 ns) as well as discarding pixels with intensity values below the average background noise floor. The threshold values of 5 ns and 17 ns for NADH and PPIX, respectively, were chosen based on the highest fluorescence lifetimes expected. For NADH, the average fluorescence lifetime is always given by a ratio of free and bound NADH, thus, it is always shorter than the fluorescence lifetime of pure enzyme bound NADH^25^. The values of bound NADH are mostly in the range of 1–4 ns^25,53^, thus, a threshold set at around 5 ns seemed reasonable. On the other hand, pure PPIX in solvent has a fluorescence lifetime of around 16 ns^21,38^ which should be the maximum value observable. As the tissue pieces differed in size we applied a normalization step to assure that every sample is contributing equally to the average lifetime. This normalization step consisted in automatically choosing 100 random pixels within the segmented lifetime maps for each sample and extracting the lifetime values for NADH and PPIX only from these points. Thereby, each sample had 100 representative fluorescence NADH and PPIX lifetime values for further statistical analysis regardless of the original physical size of the tissue.

### Statistical analysis

For each sample we averaged the 100 randomly selected points and labeled this value with the corresponding tissue type, PPIX fluorescence visibility and tumor entity/grade. We then computed the median as well as the quantiles at 25% and 75% for all unique labels and performed two-tailed Mann–Whitney U-tests (α = 5%) between groups to check for statistical significance. The choice of a non-parametric test was based on the low number of samples in certain groups.

### Classification

We additionally used our NADH and PPIX fluorescence lifetimes and fed them to a machine learning based classifier (Classification learner app, MATLAB) to predict the tumor entity/grade as well as the tissue type. As our data mainly consisted of high grade gliomas and very few low grade samples we opted for random under-sampling (RUS) boosted decision trees^54^ which are designed to handle class imbalance. Here, we used the full 100 randomly selected fluorescence lifetimes over each sample to increase the number of observations leading to a total 4200 lifetime value pairs for NADH and PPIX fluorescence. The classifier was trained using a fivefold cross-validation where the partition was patient unspecific, meaning multiple samples from a single patient are treated equally as a patient with a single sample. Although this approach tends to over-fit due to the presence of correlated data of the same source in both the training and test data set, we accepted this, as otherwise it would have resulted in only a few training samples for the smallest classes.

### Phasor plot analysis

For the phasor analysis we analyzed the raw data from our fluorescence lifetime measurements to obtain the fluorescence modulation depth and the phase delay relative to the excitation. The modulation depth is the normalized amplitude ratio between the excitation and fluorescence light. These two values were then combined to compute the phasors^36^ where the modulation corresponds to the length of the vector and the phase delay to the angle relative to the x-axis. If the fluorescence decay is mono-exponential all phasors lie on a single point on a semi-circle with the same radius or modulation depth. A bi-exponential decay is characterized by a line crossing the semi-circle at the short and long fluorescence lifetime components. Although the phasor analysis is a powerful tool to interpret complex fluorescence decays, it must be noted that a single fluorescence lifetime can only be attributed to phasors which lie on the semi-circle. While quantitative phasor analysis of NADH has recently been shown^53^, we only performed a qualitative analysis of the fluorescence decay based on the phasor plots.

## Data Availability

The fluorescence intensity and lifetime maps as well as data analysis scripts generated during the current study are available from the corresponding author on reasonable request.
